# New advances in noninvasive screening technology for colorectal cancer

**DOI:** 10.1002/mco2.70050

**Published:** 2025-01-08

**Authors:** Ning Zhu, Ziyan Tong, Ying Yuan

**Affiliations:** ^1^ Department of Medical Oncology, The Second Affiliated Hospital Zhejiang University School of Medicine Hangzhou China; ^2^ Cancer Institute, Key Laboratory of Cancer Prevention and Intervention, Ministry of Education, The Second Affiliated Hospital Zhejiang University School of Medicine Hangzhou China; ^3^ Zhejiang Provincial Clinical Research Center for CANCER Hangzhou China

1

Recently, two clinical studies on noninvasive screening tools for colorectal cancer (CRC) were published in *the New England Journal of Medicine*: the BLUE‐C study based on a next‐generation multitarget stool DNA test,^1^ and the ECLIPSE study based on a cell‐free DNA (cfDNA) blood‐based test.^2^

The currently available traditional screening methods for CRC include risk questionnaires, fecal immunochemical tests (FIT), and colonoscopy. Generally, colonoscopy with pathological biopsy is widely regarded as the gold standard for CRC diagnosis, achieving a diagnostic accuracy exceeding 95% for CRC and a sensitivity ranging from 89.1% to 94.7% for advanced adenomas.^3^ However, colonoscopy is an invasive procedure with potential risks of bleeding and perforation, while its demanding bowel preparations may lead to patient refusal. It is urgent to develop efficient noninvasive screening tools to improve patient adherence and avoid unnecessary colonoscopy.

The BLUE‐C study was conducted based on a next‐generation multitarget stool DNA testing tool (Cologuard Plus), which retained hemoglobin level assessment from its predecessor (Cologuard), and further included four genes: *LASS4*, *LRRC4*, *PPP2R5C*, and *ZDHHC1*. These genes, known as methylated DNA markers (MDMs), enable the identification of diseased tissues based on their differential methylation status. The MDMs included in Cologuard Plus are derived from various machine learning algorithms, culminating in the optimal diagnostic model trained on multiple candidates. The study enrolled 20,176 participants aged over 40 with CRC at 186 centers in the U.S. Results demonstrated that, compared with traditional FIT, the tool exhibited higher screening sensitivity for both CRC (93.9% vs. 67.3%) and advanced precancerous lesions (APL) (43.4% vs. 23.3%), but lower specificity for advanced colorectal neoplasia (ACN) (90.6% vs. 94.8%).

In 2014, Imperiale et al.^4^ marketed the first‐generation multitarget stool DNA test (Cologuard) based on the results of the DEEP‐C study (Figure 1A,C). Since then, the efficiency of Cologuard has been demonstrated across various countries (Figure 1A,C). Furthermore, it spurred the development of more screening tools utilizing remaining MDMs, such as *SDC2* in China (Figure 1A,C). However, these studies were conducted on a more limited scale.

**FIGURE 1:**
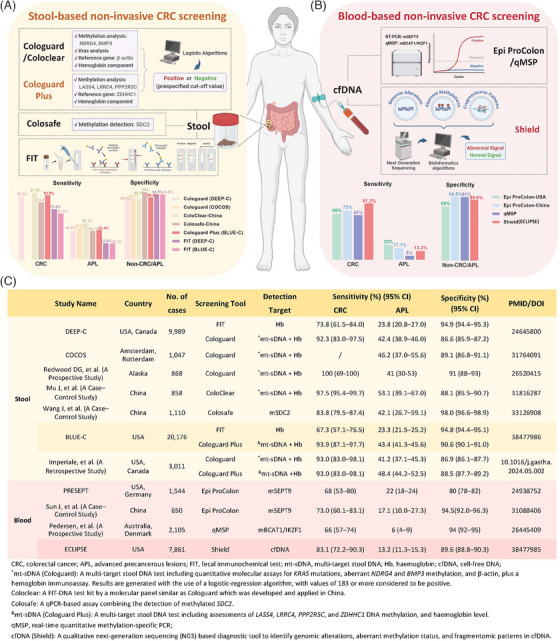
Noninvasive screening tools for colorectal cancer. (A) Stool‐based noninvasive screening tools for colorectal cancer. (B) Blood‐based noninvasive screening tools for colorectal cancer. (C) Summary and comparison of noninvasive screening tools for colorectal cancer.

Encouragingly, the BLUE‐C study demonstrated superior outcomes. Cologuard Plus exhibited higher specificity for ACN (88.5% vs. 86.9%), and increased sensitivity for APL (48.4% vs. 41.2%) compared to Cologuard (Figure 1A,C). Furthermore, although international guidelines typically recommend CRC screening starting at age 45, there exists a notable trend toward younger onset. Notably, some countries like China, propose CRC screening for individuals aged 40 to 74, alignment better with emerging epidemiological patterns and cost‐effectiveness considerations. In light of this, the BLUE‐C study encompassed a larger cohort and lowered the screening age from 45‐ to 40 years old, representing a meaningful enhancement for CRC screening. Overall, Cologuard Plus holds promise as a convenient and effective alternative as well as serves as a preliminary tool to further improve colonoscopy quality for CRC diagnosis.

However, the high cost may largely hinder the widespread adoption of stool‐based screening technology. Moreover, as highlighted in the BLUE‐C study, this method exhibited low specificity that gradually decreased with age. Such false positives may lead to unnecessary colonoscopies on elderly patients, increasing risks of bleeding and perforation while aggravating psychological and economic burdens. Future studies should focus on improving screening specificity without compromising accuracy and sensitivity to minimize healthcare resource wastage and rationalize economic costs.

In addition to stool‐based noninvasive CRC screening, blood‐based screening has undergone continuous innovation. It is well‐established that cfDNA often contains tumor‐associated mutations and epigenetic alterations that serve as valuable biomarkers for early detection. The ECLIPSE study utilized a qualitative diagnostic tool (Shield, Guardant Health) based on next‐generation sequencing, which enables the comprehensive identification of genomic alterations, aberrant methylation status, and fragmentation patterns in cfDNA from plasma. It employs advanced bioinformatics algorithms to pinpoint CRC‐associated signals, with the results aggregated into a binary outcome (abnormal or normal). The study enrolled 7861 participants aged between 45 and 84 years from 265 centers in the U.S. Results revealed that Shield showed a sensitivity of 83.1% for CRC and 13.2% for APL, with a specificity of 89.6% for ACN.

Prior to this study, the *SEPT9* gene methylation test (Epi proColon) has been extensively scrutinized as a beneficial tool for CRC screening, demonstrating notable sensitivity and specificity in the PRESEPT study (Figure 1B,C).^5^ Moreover, other MDMs in the bloodstream have exhibited potential as diagnostic tools for CRC screening, such as dual gene methylation of *BCAT1/IKZF1* (Figure 1B,C), which requires further large‐scale clinical validation.

The Shield tool, which holds the ability to detect a wide array of genetic variations including but not limited to DNA methylations and mutations, has shown promising progress in blood‐based noninvasive CRC screening. However, its ability to detect APL remains limited. Compared with Epi proColon, it demonstrated higher sensitivity (83.1% vs. 68%) and specificity (89.6% vs. 80%) for CRC screening, but significantly lower sensitivity for APL (13.2% vs. 22%; Figure 1B,C). Additionally, when compared side‐by‐side with other noninvasive screening tools, the Shield tool showed improved sensitivity for CRC over FIT (67.3%) but was slightly inferior to Cologuard Plus (93.9%). When considering specificity for ACN and sensitivity for APL, both FIT (94.8% and 23.3%) and Cologuard Plus (90.6% and 43.4%) outperformed the Shield tool.^1^

Taken together, the Shield tool successfully met the FDA criteria for sensitivity and specificity in CRC screening, despite certain limitations compared with stool‐based tests. Undoubtedly, it could offer a more comprehensive and convenient alternative that encourages more participation in CRC screenings. Blood‐based DNA tests might be more appropriate for individuals with benign bleeding lesions who are unsuitable for stool‐based tests. They can also serve as a secondary approach for those who decline stool tests yet wish to engage in screening.

As for the limitations, it is undeniable that Shield demonstrated lower sensitivity and specificity compared to stool‐based screening technologies. Combining other blood analytes such as cell‐free RNA and exosomes, along with improving cfDNA detection capabilities, may further enhance its detection efficiency. Additionally, the blood collection standard (30–80 mL) mentioned in the ELIPSE study, may reduce patient adherence and present challenges for clinical use. Therefore, future studies should focus on enhancing screening sensitivity through technological refinement, while fully considering ways to lower costs and reduce sampling volume requirements. Notably, compared with the BLUE‐C study, the smaller participant number in the ECLIPSE study may introduce conclusion bias. Moreover, there exist differences in the composition of the clinical validation cohort compared to typical screening populations, which necessitates further validation to confirm the applicability of these results to a broader population, ensuring their accuracy and reliability.

Overall, these noninvasive tests for CRC can significantly decrease unnecessary colonoscopies, but the costs and acceptance of new technologies must be carefully considered for both screening tools. We are optimistic about the potential for further advancements in reducing costs while maintaining accuracy. Innovations in sequencing technology and economies of scale are anticipated to reduce costs. Moreover, improved screening strategies targeting high‐risk groups could further improve cost‐effectiveness.

For patients, affordable, convenient, and accessible noninvasive screening technology with reliable results will undoubtedly increase their willingness to participate in early screening. In the future, multidimensional rationalization in terms of testing technology, cost, and level of clinical evidence will probably be the main goal in designing and evaluating CRC screening technologies.

## AUTHOR CONTRIBUTIONS

Ning Zhu and Ziyan Tong conceived and drafted the manuscript. Ying Yuan provided guidance for the manuscript. All authors have read and approved the final manuscript.

## CONFLICT OF INTEREST STATEMENT

The authors declare no conflict of interest.

## ETHICS APPROVAL

Not Applicable.

## Data Availability

Not Applicable.
